# Pulmonary Embolism as the First Manifestation of Multiple Myeloma

**DOI:** 10.1155/2013/236913

**Published:** 2013-09-18

**Authors:** N. Vallianou, V. Lazarou, J. Tzangarakis, R. Barounis, E. Sioula

**Affiliations:** Internal Medicine Department, Evangelismos General Hospital, 45-47 Ipsilantou Street, 10676 Athens, Greece

## Abstract

Multiple myeloma is considered a hypercoagulable state due to several mechanisms such as the increased IL-6 and immunoglobulins production, the defective fibrinolytic mechanism, and the acquired resistance to activated protein C that are involved in the pathogenesis and clinical futures of the disease. We describe a case of a female patient who presented to the hospital with pulmonary embolism as the first manifestation of the hypercoagulability of multiple myeloma.

## 1. Introduction

Venous thromboembolic disease has been well established as a major complication of myeloma therapy [1–3]. Also, thrombosis is recognized as the most frequent complication of malignant diseases. Patients with multiple myeloma are predisposed to an increased incidence of venous thromboembolic disease and often have additional risk factors from prolonged bed rest, back pain, and the presence of amyloidosis and hyperviscosity, all of which contribute to hypercoagulability [4]. Herein, we describe the case of a patient who presented with pulmonary embolism and was subsequently found to have multiple myeloma.

## 2. Case Presentation

A forty-two-year-old female patient came to the hospital due to dyspnea and swelling of her left leg, which had started two days before her admission.

On physical examination, she had tachypnea and tachycardia, while she had crackles on auscultation of the thorax. Her left leg was edematous from the hip down to the ankle. A triplex of the venous vasculature of the left leg revealed thrombosis of the left femoral venous, while the vasculature of the right leg was normal. Due to tachypnea with hypoxia, she underwent a spiral thoracic CT scan that confirmed the diagnosis of pulmonary embolism.

The ECG showed sinus tachycardia. From the laboratory tests, she had a very high ESR (120 mm/h), an albumin to globulins ration of 0.86, serum albumin: 2.7 g/dL, *β*2-microglobulin: 2,076 *μ*g/L, while IgG measurement was 4,490 mg/dL, IgM was 36.9 mg/dL, IgA was 40.5 mg/dL, and there was a band formed in the place of gamma globulins in serum electrophoresis. Because of those laboratory values, she underwent a bone marrow aspiration, which confirmed the presence of 70% myeloma cells in the bone marrow. Thrombophilia laboratory tests were normal. The patient was initially placed on low-molecular-weight heparin (LMWH) and afterwards on warfarin for six months. After having been hospitalized for pulmonary embolism for 15 days, she started therapy with bortezomib and she remains well until today.

## 3. Discussion

Several different mechanisms have been described as possible causative factors for venous thromboembolic disease in patients with multiple myeloma. High levels of IL-6 are associated with the coagulation cascade activity [5]. The presence of abnormal levels of immunoglobulins alters the network structure of gels formed from purified fibrinogen. The fibrin fibers that compose these gels have been shown by electron microscopy to be much thinner than normal. Moreover, plasma clots comprising thin, small-diameter fibrin fibers are found to be relatively resistant to plasmin degradation [6, 7]. Also, acquired activated protein C resistance (APC-R) was recognized to be common in patients with cancer and recently especially among patients with multiple myeloma [2, 8–10]. Multiple myeloma patients also have decreased fibrinolytic activity because of increased PAI-1 (plasminogen activator inhibitor-1) activity, which is positively related to elevated IL-6 levels [11]. Apart from having an increased risk of pulmonary embolism due to immunomodifying agents that are included in multiple myeloma therapy, patients with newly diagnosed multiple myeloma, that is prior to having received therapy, seem to be at increased risk for venous thromboembolic disease and pulmonary embolism [12]. Our patient presented to the hospital with pulmonary embolism and she was diagnosed as having multiple myeloma, stage II according to the ISS. Thrombophilia tests were normal, that is, testing for antithrombin III, protein C, protein S, lupus anticoagulant-1, while the molecular testing for FV Leiden, prothrombin gene, and MTHFR were negative too. The hypercoagulability associated with multiple myeloma, which is explained by the above-mentioned mechanisms, could have been responsible for her presentation with pulmonary embolism. Further studies are needed to access the risk for pulmonary embolism in multiple myeloma patients prior to the administration of immunomodifying agents and/or chemotherapy.
